# Consumer Values, Attitudes, and Behavior towards Plant-Based Alternatives

**DOI:** 10.3390/foods13162561

**Published:** 2024-08-16

**Authors:** Cho-I Park, Young Namkung

**Affiliations:** 1 Smart Tourism Education Platform, Kyung Hee University, 26 Kyungheedae-ro, Dongdaemun-gu, Seoul 02447, Republic of Korea; agnes.cho-i@khu.ac.kr; 2 College of Hotel & Tourism Management, Kyung Hee University, 26 Kyungheedae-ro, Dongdaemun-gu, Seoul 02447, Republic of Korea

**Keywords:** plant-based alternatives (PBAs), value–attitude–behavior (VAB) framework, consumer values, attitudes, intentions, behavior

## Abstract

This study investigated the impact of consumer values and attitudes to consuming plant-based alternatives (PBAs), using the value–attitude–behavior framework. The research model and hypotheses were tested using a two-step approach to structural equation modeling on 392 responses collected from PBA consumers through a research company in Korea in November 2023. The results indicated that environmental consciousness and health consciousness significantly affected attitudes toward PBAs. Also, positive attitudes toward PBAs were critical for the formation of repurchase intentions for PBAs and PBA restaurant visit behavior. Our study contributes to both academics and PBA practitioners by showing how consumer values are associated with attitudes, PBAs repurchase intentions, and PBAs restaurant visits.

## 1. Introduction

Meat production accounts for 57% of global greenhouse gas emissions from all food production, which is twice that from plant-based food production [1]. Switching to a plant-based diet could reduce greenhouse gas emissions by up to 49% globally [2]. The foodservice industry is recognized as a significant contributor to global climate change in terms of food production and consumption [3]. Accordingly, major quick-service restaurant chains such as Burger King and Subway have added ‘meat-like’ menus [4]. Starbucks, a global coffee brand, is committed to sustainability by adding and expanding its plant-based menu across its beverage and food offerings, aiming to reduce its carbon footprint by 50% [5].

Health consciousness and environmental sustainability have often been discussed as important factors in developing plant-based alternatives (PBAs) [6,7]. Consumer research also has reported that the biggest market drivers for PBA purchases are health and environmental consciousness. A recent study by Bakr, Al-Bloushi, and Mostafa (2023) highlighted the importance of environmental consciousness in consumers’ positive attitudes and purchase intentions with regard to plant-based meat alternatives [8]. In addition, consumers’ consciousness of health issues is the strongest factor in their intention to purchase PBAs [9]. Therefore, consumer awareness is a criterion that can be used to select food and justify food-related behavior [10]. Shifting towards a plant-based diet, from the consumers’ point of view, has positive health benefits [11,12] and may also significantly improve environmental sustainability [13]. In addition, from a business perspective, understanding consumers’ attitudes toward products and their purchasing behavior, can help businesses to develop insights on sustainable marketing models in the market [14].

In the hospitality domain, numerous studies have identified the importance of the ‘Value-Attitude-Behavior (VAB)’ theory in explaining consumers’ sustainable behavior [15,16]. This theory, propounded by Homer and Kahle (1988) [17], focuses on consumers’ personal values influencing their decision-making processes [18]. The extant literature on consumer behavior suggests that both altruistic and egoistic values play an important role in determining eco-friendly behavior [19,20]. Health consciousness is an egoistic value that benefits the individual, while environmental consciousness is an altruistic value because it helps the environment rather than the individual [21]. In accordance with recent research on PBAs [8,22], this study considers health consciousness as an egoistic value and environmental consciousness as an altruistic value.

Based on an extensive literature review, we found that several studies have been conducted using consumers’ purchase intention as a final variable. Although the recent increase in PBAs in the foodservice industry has received considerable attention, the existing literature has not yet investigated the influence of the preceding variables on customers’ visits to restaurants featuring PBAs. Furthermore, most of the research on PBAs has focused solely on plant-based meat alternatives. Also, prior research noted an ‘intention-behavior gap’ [23]. Does this gap really exist?

Given this research gap, this study aimed to (1) investigate the impact of consumers’ values (health consciousness, environmental consciousness) on their attitudes toward PBAs and (2) examine the relationship between attitudes, PBAs repurchase intentions, and PBAs restaurant visits. To achieve the research purpose, we first present the conceptual background and develop the hypotheses. Then, we describe the research methodology and present the data analysis and results. Finally, we present the theoretical and managerial implications of the study results and discuss the limitations of the study and directions for future research.

## 2. Literature Review and Hypothesis Development

### 2.1. Literature Review

#### 2.1.1. Plant-Based Alternatives (PBAs)

Plant-based alternatives (PBAs) are foods manufactured using proteins extracted from plants. They include plant-based meat alternatives, plant-based milk alternatives, plant-based egg alternatives, and plant-based beverages [24]. Among them, plant-based meat alternatives and plant-based milk alternatives account for an overwhelming proportion of the global alternative food market [24]. The global market size of plant-based meat alternatives is expected to reach 4.04 billion US dollars in 2029. China is the largest plant-based meat alternative market, followed by the US and the UK [25]. Additionally, in 2024, the Asia Pacific region had the highest value of plant-based milk alternatives in the world, amounting to approximately 9.9 billion U.S. dollars, followed by North America and Europe [26]. Research shows that the PBAs consumers tried the most in South Korea in 2022 were plant-based milk alternatives, while only around one in four people had tried plant-based meat alternatives [27]. Accordingly, in this study, considering the current status of the PBA market in Korea, plant-based meat alternatives and plant-based milk alternatives were defined as PBAs and selected as research subjects. Plant-based meat alternatives are made from proteins extracted from plants such as soy and peas [28]. Additionally, plant-based milk alternatives, which are a substitute for cow’s milk, are mixtures of water and plant ingredients extracted from legumes, nuts, cereals, or pseudocereals [29,30]. Therefore, PBAs have nutritional, sensory, and technological properties similar to animal-based products such as meat and milk [31,32]. Both health and environmental consciousness are frequently mentioned as major reasons for developing PBAs [6,33]. Consumer studies report that PBAs offer health benefits and contribute to environmental sustainability. As consumers recognize that reducing meat-based foods in diets can be beneficial for health and the environment, interest in PBAs is also growing [34]. Accordingly, food enterprises are paying more attention to the growth potential of PBAs, and the number of vegetarian restaurants in the foodservice industry is also increasing [35].

#### 2.1.2. Value–Attitude–Behavior (VAB) Framework

The VAB theory, propounded by Homer and Kahle (1988) [17], suggests that consumers’ values are significant in forming attitudes, which in turn result in behavior [18]. Values are defined as the ways in which people are guided to behave [36]. Attitude refers to an individual’s consistent tendency to react favorably or unfavorably toward the objects in question [37], and is based on more constant and enduring values. An individual’s behavior is determined by their values and attitudes [17]. This means that individuals engage in particular behaviors based on the priority of perceived values [38]. Therefore, the VAB theory postulates that an individuals’ values and attitudes are precursors to intention and behavior [39]. Consequently, the hierarchical structure linking values, attitudes, intention, and behavior emphasizes that values are the fundamental factors: based on values and attitudes, an individual manifests an actual behavior [17].

### 2.2. Hypothesis Development

#### 2.2.1. Values (Health Consciousness, Environmental Consciousness) and Attitudes toward PBAs

Health consciousness means evaluation of one’s readiness to engage in healthy behavior [40] and is an egoistic value that benefits the individual [21]. Consuming PBAs can reduce health risks including cardiovascular diseases [11], cancer, and diabetes, through their antioxidant activities [41]. Consumers tend to perceive plant-based foods as healthy [42]. Therefore, health-conscious consumers are more likely to reduce their intake of animal-based food consumption and increase their intake of plant-based foods [43]. Kumar (2021) reported that consumers’ attitudes toward products perceived as beneficial to personal health are significantly influenced by health consciousness [44]. In addition, some studies have highlighted that PBAs are chosen not only by people with health consciousness but also by people with health problems [28,45]. The results showed that health consciousness may have a positive effect on attitudes toward PBAs. Hence, we proposed the following hypothesis.

**Hypothesis** **1.** *Consumers’ health consciousness has a positive impact on attitudes toward PBAs*.

Consumers’ environmental consciousness has been raised as one of the main reasons for consumers to choose PBAs when purchasing foods [46]. This is a prerequisite for eco-friendly consumption [47,48], and is an altruistic value because it helps the environment rather than the individual [21]. Alibeli and Johnson (2009) defined environmental consciousness as the degree to which an individual recognizes and tries to solve environmental problems [49]. This concept also includes attitudes and evaluations of one’s own and others’ environment-related behavior, and concerns about environmental issues [50]. Profeta et al. (2021) presented environmental consciousness as one of the key values for choosing environmentally friendly foods and demonstrated that it had a positive effect on attitudes toward PBAs [46]. In addition, Bakr, Al-Bloushi and Mostafa (2023) found that environmental consciousness positively impacts attitudes toward plant-based meat alternatives [8]. Therefore, it can be assumed that consumers choosing PBAs based on environmental consciousness have positive attitudes toward PBAs. Hence, we proposed the following hypothesis.

**Hypothesis** **2.** *Consumers’ environmental consciousness has a positive impact on attitudes toward PBAs*.

#### 2.2.2. Attitudes toward PBAs and PBAs Repurchase Intention

Consumer attitudes are vital because they precede the intention to perform a specific behavior [51]. Pandey, Ritz, and Perez-Cueto (2021) established that consumers’ attitudes toward products influence their consumption intention [52]. Consistent with the research finding [9] that attitudes have the strongest effect on behavioral intention, Bakr, Al-Bloushi, and Mostafa (2022) found that positive attitudes significantly improved consumers’ behavioral intentions relating to [8]. In other words, a favorable attitude toward PBAs can have a positive effect on PBA repurchase intentions. Hence, we proposed the following hypothesis.

**Hypothesis** **3.** *Attitudes toward PBAs have a positive impact on PBA repurchase intentions*.

#### 2.2.3. Attitudes toward PBAs and PBA Restaurant Visits

The link between attitudes and actual behavior varies across studies. Applying the VAB theory in the context of eco-friendly hotels, scholars have argued that positive attitudes actually increase environmentally friendly behavior [16]. Previous studies contended that attitudes are among the strongest drivers of actual behavior [53]. However, some researchers have argued that consumers’ attitudes do not always precede behavior [54,55]. Although there is a mismatch between consumers’ positive attitudes and their corresponding actual behavior, we assume that consumers’ attitudes toward PBAs may result in positive PBA restaurant visits. For this reason, the following hypothesis was proposed.

**Hypothesis** **4.** *Attitudes toward PBAs have a positive impact on PBA restaurant visits*.

#### 2.2.4. PBA Repurchase Intention and PBA Restaurant Visits

Although the prevailing opinion is that consumers’ behavioral intention is a critical factor in actual behavior [56], some studies in consumption contexts have not established a causal relationship between intention and behavior [23,57]. In contrast, Agag and Colmekcioglu (2020) found that consumers’ behavioral intentions had a positive effect on actual behavior [58]. Similarly, a study verified that consumers’ intention to consume PBAs is an influential factor in actual consumption [52]. Supporting these results, Chakraborty et al. (2022) noted that purchase intention and buying behavior had a positive association [59]. Therefore, this study assumed that consumers who have a high intention to continue purchasing will also engage in PBA restaurant visit behavior.

**Hypothesis** **5.** *PBA repurchase intention has a positive impact on PBA restaurant visits*.

**Figure 1:**
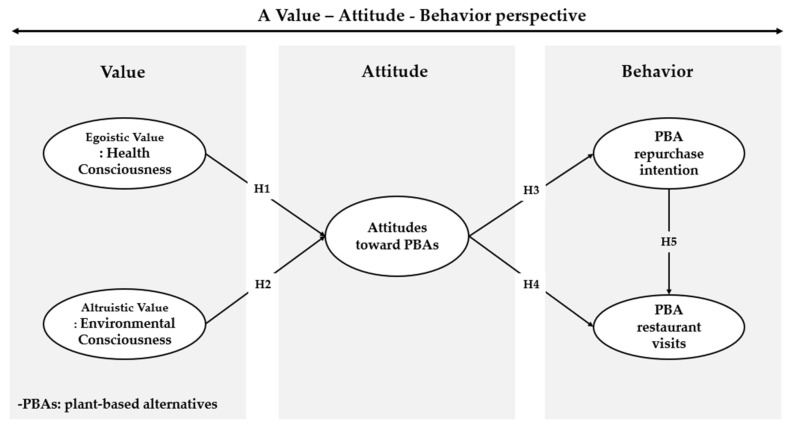
Research model.

Figure 1 presents the research model and hypotheses.

## 3. Material and Methods

### 3.1. Data Collection and Sampling Methods

A preliminary study was conducted with 45 students with experience of consuming PBAs. Based on feedback from this pilot test, ambiguous words and sentences were modified for the final version of the main survey questionnaire. Participants were limited to those over 20 years old who had experienced purchasing PBAs within the last 3 months. According to a prior study, the number of vegetarians in Korea was found to be as low as 1%, with very few strict vegans. In addition, the vegetarian population appeared to be evenly distributed from those in their 20s to those in their 60s, so adults in their 20s or older who had experience purchasing PBAs were judged to be suitable for our research. A self-administered online survey was conducted over a week in November 2023 through a research company in Korea. Based on prior research, all scale items were modified to fit the study’s context. Health and environmental consciousness in the value dimensions were, respectively, assessed using 4 items, adopted from Hansen, Sørensen, and Eriksen (2018), Namkung and Jang (2017), Tarkiainen and Sundqvist (2005), Schlegelmilch, Bohlen, and Diamantopoulos (1996), and Yadav and Pathak (2016) [60,61,62,63,64]. Attitudes toward PBAs were measured with 3 items adopted from Fishbein and Ajzen (1977) and Chen (2007) [65,66]. PBA repurchase intention and PBA restaurant visits were measured with 3 items adopted from Zeithaml et al. (1996) [67]. All survey items were measured using a 7-point Likert scale ranging from 1 (strongly disagree) to 7 (strongly agree). Table 1 displays the demographic characteristics of the sample. The results show that 50.5% of the respondents were female and 49.5% were male. Most of the respondents were in their 20s and 30s, single (54.3%), office workers (43.6%), university students/graduates (79.1%), and Seoul residents (27.3%). In terms of PBA consumption patterns, the most common recently purchased PBAs were plant-based milk alternatives (58.7%), and the places where PBAs were purchased were offline (hypermarkets/warehouses) (43.4%).

### 3.2. Data Analysis

Data analysis was performed using SPSS 25.0 and AMOS 23.0 statistical software. First, respondents’ socio-demographic characteristics backgrounds and PBA consumption patterns were tested with SPSS 25.0. Second, to confirm the validity and reliability of the study variables, confirmatory factor analysis (CFA) was performed. Finally, the proposed hypotheses were verified using structural equation modeling (SEM). A total of 8 incomplete responses were eliminated from the original data, and 392 responses were included in the final analysis.

## 4. Results and Discussion

To evaluate the validity and reliability of the measurements, we conducted confirmatory factor analysis (CFA). As shown in Table 2, the measurement model fits the data reasonably well (χ^2^ = 279.186, df = 109, *p* < 0.001, χ^2^/df = 2.561, CFI = 0.971, NFI = 0.954, TLI = 0.964, IFI = 0.971, RMSEA = 0.063). All standardized coefficients were greater than 0.50, ranging from 0.750 to 0.950, thus confirming convergent validity as described by Hair et al. (2010) [68]. Cronbach’s alpha coefficients ranged from 0.878 to 0.950, supporting the internal consistency of items within each construct as they exceeded the reference value of 0.7 [69]. All constructs of average variance extracted (AVE) exceeded the minimum acceptable value of 0.50, ranging from 0.716 to 0.865. In addition, the values for composite construct reliability (CCR) were higher than the threshold value of 0.70, ranging from 0.883 to 0.951, supporting the convergent validity and reliability of the measures [70]. As indicated in Table 3, the AVE values were greater than the squared correlation between the constructs, which is evidence of discriminant validity [70].

Structural equation modeling (SEM) results with standardized path coefficient and t-values are provided in Table 4. The research model had acceptable indicators for the goodness of fit (χ^2^ = 313.050, df = 113, *p* < 0.001, χ^2^/df = 2.770, CFI = 0.966, NFI = 0.948, TLI = 0.959, IFI = 0.966, RMSEA = 0.067 [70]. Customers’ health consciousness (*β* = 0.310, *p* < 0.001) and environmental consciousness (*β* = 0.414, *p* < 0.001) positively influenced attitudes toward PBAs. Attitudes toward PBAs had a positive effect on repurchase intention for PBAs (*β* = 0.853, *p* < 0.001) and PBA restaurant visits (*β* = 0.318, *p* < 0.001). Finally, repurchase intention for PBAs was significantly associated with PBA restaurant visits (*β* = 0.439, *p* < 0.001). Thus, all hypotheses were supported. The proposed model assigned 40.1% to attitude, 72.8% to intention, 53.2% to behavior, demonstrating a strong predictive capacity.

The analysis indicated that values (health and environmental consciousness) significantly affected attitudes toward PBAs. Thus, our results supported hypotheses 1 and 2 and were in line with the findings from previous studies [8,9]. The results reflect the increasing consumer demand for sustainable and healthy food products. Attitudes toward PBAs were the crucial factor in creating repurchase intention for PBAs and PBAs restaurant visits. This adds to the literature on the influence of attitudes on intention [8,9] and behavior [16,71], confirming hypotheses 3 and 4, respectively. This implies that consumers’ favorable attitudes are a vital predictor of repurchase intention for PBAs and PBAs restaurant visits. We also found that PBAs repurchase intention had a significant impact on PBAs restaurant visits, confirming hypothesis 5. This is consistent with previous studies [58,59]. The research findings emphasize that values are significant predictors of attitudes towards PBAs. Additionally, in line with previous research concerning food selection behavior, attitudes were crucial in forming intentions and behaviors [71,72].

## 5. Conclusions

The present study has several theoretical implications. First, VAB appeared to be a robust theoretical framework that was applied to investigate the sequential relationship of values, attitudes, and behaviors with consumer decision-making in the PBA context. Second, this study extended the existing literature, emphasizing the link between intention and behavior. Considering the trend of major PBA manufacturers collaborating with restaurant chains and cafes (e.g., McDonald’s, Starbucks) to add PBA menus to their range [4,73], our study included PBA restaurant visiting behavior as a variable. While most previous studies have dealt with the relationship between attitude and purchase intention, this study extends the research on PBAs by finding significant associations between consumer values, attitudes, repurchase intentions for PBAs, and PBA restaurant visits.

Our findings provide practical implications for PBA brand marketers and foodservice operators. First, our study showed that health and environmental consciousness are essential factors in inducing favorable attitudes toward PBAs, which in turn influence PBAs repurchase intentions and PBA restaurant visits. Therefore, we suggest that PBA companies use the health benefits and environmental sustainability of their products as part of their marketing strategy. When promoting products, for instance, marketers can create favorable attitudes in consumers by providing consumers with practical information about the health benefits and environmental consequences of PBA consumption through their advertisements and SNSs. Second, restaurants could consider adding PBA options to their menus. Furthermore, PBA enterprises could consider partnership with restaurants to promote their products. In the case of a domestic PBA company (e.g., Maeil Dailies), they developed a plant-based beverage menu in collaboration with local cafes and promoted their own products. If restaurants use such methods as a competitive advantage, it can give consumers a novelty dining experience and serve as an opportunity to identify future markets based on consumer responses. As a result, co-developing and promoting plant-based menu items may lead to positive outcomes for both PBA enterprises and restaurants, including increased favorable attitudes, repurchase intentions, and finally restaurant visits. Third, regarding the demographic characteristics of the respondents in our study, more than half were in their 20s and 30s (59.6%), and single (54.3%). PBA companies need to segment consumers considering their influence in society and purchasing power, and it is necessary to appropriately mix PBA products, distribution channels, promotions, and prices to appeal to target consumers. Our results also showed that purchases made through offline stores accounted for the highest percentage of sales (43.4%). This may mean that offline distribution channels need to be strengthened when selling PBAs.

The current study has some limitations. First, our research data were collected from South Korea. Attitudes toward PBAs may differ in countries where meat is an important part of culinary traditions and those where it is not. For example, the US has the world’s highest meat consumption, while India has the highest percentage of vegetarians [74]. India is home to the majority of the vegans in the world, and vegetarian diets are deeply rooted in Indian cuisine [74]. In contrast, overconsumption of meat and a lack of willingness to adopt a plant-based diet are still dominant cultural patterns in most Western societies [75]. Runte et al. (2024) demonstrated cross-cultural differences in perceptions of PBAs among consumers [74]. Similarly, a prior study also reported that plant-based meat alternatives are viewed more favorably in India than in the US [10]. Food, eating, and nutrition are strongly influenced by culture [76]. Cultural aspects such as cultural capital could drive consumers’ food choices [77]. This result suggests that consumer attitudes toward PBAs may vary across cultural differences. Therefore, a follow-up study is necessary to apply our research framework to other countries where PBAs are popular and widely used, or where there are differences in cultural capital. Second, measuring actual behavior is not simple in the case of relatively novel foods such as PBAs. Accordingly, as in most previous studies, our study used behavioral intention scales to measure PBA restaurant visits. However, some research has indicated that there can be an ‘intention-actual behavior gap’ [23]. To fill this gap, we recommend that future studies use items measuring actual behavior or behavioral continuity. Third, considering the revenue growth rate in the PBA market globally, it is necessary to extend research to include various PBA products (such as plant-based egg alternatives or plant-based desserts). For instance, the global vegan desserts market is expected to grow at a CAGR (compound annual growth rate) of 10% from 2021 to 2027, so research on this subject will be meaningful. Fourth, further research should investigate the influence of other variables related to PBA consumption. Product attributes (sensory appeal, price, food safety), situational factors (product availability) and food-related personality traits (food neophobia, food neophilia) can be considered, which have been mentioned as major determinants of consumer purchasing behavior. In doing so, consumer purchasing behavior will be more effectively understood. Finally, this study used cross-sectional data to examine the relationship between consumption values, attitudes, behavioral intention, and behavior relating to PBAs. However, cross-sectional studies only provide static results from those perspectives [78]. In addition, attitudes toward foods could change and improve as familiarity with PBAs increases over time [8]. Therefore, conducting longitudinal studies would be recommended for future research to explore changes in attitude, behavioral intention, and behavior relating to PBA consumption values over time.

## Figures and Tables

**Table 1:** Demographic characteristics of the sample (*n* = 392).

Demographic Characteristics	*n*	%	Demographic Characteristics	*n*	%
**Gender**			**Marital Status**		
Male	194	49.5	Single	213	52.0
Female	198	50.5	Married	177	48.0
			Others	2	0.5
**Age**			**Occupation**		
20–29	117	29.8	Student	35	8.9
30–39	117	29.8	Office worker	171	43.6
40–49	98	25.0	Service worker	32	8.2
Avove 50	60	15.3	Public officer	25	6.4
**Education**			Professional worker	32	8.2
High school graduate or below	42	10.7	Self-employed	28	7.1
College/University	310	79.1	Housewife	45	11.5
Graduated school and above	40	10.2	Others	24	6.1
**Monthly household income**			**Household size**		
≤1999 thousand won	50	12.8	One person (self)	85	21.7
2000–3999 thousand won	182	46.4	Two persons	71	18.1
4000–5999 thousand won	78	19.9	Three persons	96	24.5
6000–7999 thousand won	51	13.0	Four persons	118	30.1
≥8000 thousand won	31	7.9	Five persons or more	22	5.6

**Table 2:** Results confirmatory factor analysis.

Constructs			M ± S.D.	Standardized Loading	AVE	CCR	Cronbach’s α
V	HC	I choose food carefully for my health.	5.10 ± 1.17	0.813	0.730	0.915	0.915
		I think about my health issues often.	5.29 ± 1.19	0.865			
		I pay a lot of attention to my health.	5.20 ± 1.18	0.880			
		I try to choose healthy foods.	5.29 ± 1.19	0.859			
	EC	I am interested in environmental protection.	4.97 ± 1.29	0.872	0.742	0.920	0.897
		I buy products that have a less harmful impact on the environment.	4.70 ± 1.29	0.887			
		I tend to be conscious of environmental issues when purchasing products.	5.43 ± 1.21	0.896			
		I buy products that have less impact on the environment if the products are otherwise the same.	5.15 ± 1.30	0.786			
A	ATT	Choosing PBAs was a wise decision.	4.68 ± 1.17	0.882	0.716	0.883	0.878
		I am satisfied with choosing to eat PBAs.	4.81 ± 1.14	0.899			
		PBAs are trustworthy.	4.96 ± 1.02	0.750			
B	PRI	I plan to continue purchasing PBAs in the future.	4.89 ± 1.25	0.793	0.761	0.905	0.897
		I would like to spread information about PBAs.	4.59 ± 1.25	0.896			
		I would like to share product information about PBAs with my friends.	4.59 ± 1.30	0.922			
	PRV	If I have the chance, I will visit a PBAs restaurant.	4.33 ± 1.44	0.925	0.865	0.951	0.950
		I will visit PBAs restaurants.	4.21 ± 1.44	0.950			
		I would like to visit a restaurant if it has a PBAs menu.	4.26 ± 1.41	0.915			

χ^2^ = 279.186, df = 109, *p* < 0.001, χ^2^/df = 2.561, CFI = 0.971, NFI = 0.954, TLI = 0.964, IFI = 0.971, RMSEA = 0.063. **HC**: health consciousness, **EC**: environmental consciousness, **ATT**: attitudes toward PBAs, **PRI**: PBA repurchase intention, **PRV**: PBA restaurant visit.

**Table 3:** Correlations and discriminant validity.

	HC	EC	ATT	RI	RV
**HC**	**0.730 ^a^**				
**EC**	0.520 **^b^** (0.270) **^c^**	**0.742**			
**ATT**	0.511 (0.261)	0.533 (0.284)	**0.716**		
**PRI**	0.492 (0.207)	0.603 (0.364)	0.834 (0.696)	**0.761**	
**PRV**	0.369 (0.136)	0.512 (0.262)	0.679 (0.461)	0.709 (0.503)	**0.865**

**Note**: **^a^** Average variance extracted (AVE); **^b^** correlation (R); **^c^** squared correlation (R^2^).

**Table 4:** Results of hypothesis testing.

Hypothesized Path		Standardized Estimate	C.R.	P	Results
H1	HC → ATT	0.310	5.600 ***	0.000	Supported
H2	EC → ATT	0.414	7.433 ***	0.000	
H3	ATT → PRI	0.853	16.265 ***	0.000	
H4	ATT → PRV	0.318	3.419 ***	0.000	
H5	PRI → PRV	0.439	4.697 ***	0.000	

χ^2^ = 313.050, df = 113, *p* < 0.001, χ^2^/df = 2.770, CFI = 0.966, NFI = 0.948, TLI = 0.959, IFI = 0.966, RMSEA = 0.067. *** *p* < 0.001. Total variance explained (R^2^): R^2^ for attitude toward PBAs = 0.401; R^2^ for PBA repurchase intention = 0.728; R^2^ for PBA restaurant visit **=** 0.532.

## Data Availability

The data presented in this study are available on request from the corresponding author due to ethical considerations.
